# Analysis of *in vitro* secretion profiles from adipose-derived cell populations

**DOI:** 10.1186/1479-5876-10-172

**Published:** 2012-08-22

**Authors:** Sinead P Blaber, Rebecca A Webster, Cameron J Hill, Edmond J Breen, Donald Kuah, Graham Vesey, Benjamin R Herbert

**Affiliations:** 1 Department of Chemistry and Biomolecular Sciences, Macquarie University, Office 256, Building E8C, Balaclava Rd, North Ryde, NSW 2109, Australia; 2 Regeneus Ltd, Gordon, NSW, Australia; 3 Australian Proteome Analysis Facility, Macquarie University, North Ryde, NSW, Australia; 4 Sydney Sportmed Specialists, Sydney, NSW, Australia

**Keywords:** Adipose tissue, Adipose-derived stem cells, Stromal vascular fraction, Cytokines, Growth factors, Bio-Plex

## Abstract

**Background:**

Adipose tissue is an attractive source of cells for therapeutic purposes because of the ease of harvest and the high frequency of mesenchymal stem cells (MSCs). Whilst it is clear that MSCs have significant therapeutic potential via their ability to secrete immuno-modulatory and trophic cytokines, the therapeutic use of mixed cell populations from the adipose stromal vascular fraction (SVF) is becoming increasingly common.

**Methods:**

In this study we have measured a panel of 27 cytokines and growth factors secreted by various combinations of human adipose-derived cell populations. These were 1. co-culture of freshly isolated SVF with adipocytes, 2. freshly isolated SVF cultured alone, 3. freshly isolated adipocytes alone and 4. adherent adipose-derived mesenchymal stem cells (ADSCs) at passage 2. In addition, we produced an ‘*in silico*’ dataset by combining the individual secretion profiles obtained from culturing the SVF with that of the adipocytes. This was compared to the secretion profile of co-cultured SVF and adipocytes. Two-tailed t-tests were performed on the secretion profiles obtained from the SVF, adipocytes, ADSCs and the ‘*in silico*’ dataset and compared to the secretion profiles obtained from the co-culture of the SVF with adipocytes. A p-value of < 0.05 was considered statistically different. To assess the overall changes that may occur as a result of co-culture we compared the proteomes of SVF and SVF co-cultured with adipocytes using iTRAQ quantitative mass spectrometry.

**Results:**

A co-culture of SVF and adipocytes results in a distinct secretion profile when compared to all other adipose-derived cell populations studied. This illustrates that cellular crosstalk during co-culture of the SVF with adipocytes modulates the production of cytokines by one or more cell types. No biologically relevant differences were detected in the proteomes of SVF cultured alone or co-cultured with adipocytes.

**Conclusions:**

The use of mixed adipose cell populations does not appear to induce cellular stress and results in enhanced secretion profiles. Given the importance of secreted cytokines in cell therapy, the use of a mixed cell population such as the SVF with adipocytes may be considered as an alternative to MSCs or fresh SVF alone.

## Background

Until recently, adipose tissue was considered not only to be a passive storage organ for triglycerides and therefore primarily an energy source, but also a structure which can provide thermal and mechanical insulation. However, a number of key discoveries identified the importance of adipose tissue as an endocrine organ with major roles in a wide variety of processes in the body. It is now known that adipose tissue expresses and secretes an array of bioactive factors, which act at the autocrine, paracrine and/or endocrine level. These bioactive factors are involved in a diverse set of functions including lipid metabolism, reproduction, angiogenesis, insulin sensitivity, appetite and energy balance, blood pressure regulation and inflammation [1].

Adipose tissue is a highly vascularized organ containing a dense network of capillary beds surrounding mature adipocytes. Associated with these capillary beds are a number of different cell types. Using collagenase, the connective tissue can be broken down, thereby releasing the cells. Subsequent centrifugation results in floating adipocytes, and pelleted cells, termed the stromal vascular fraction (SVF). The SVF contains high numbers of T regulatory cells and macrophages, as well as endothelial cells and smooth muscle cells [2,3]. Additionally, the SVF from adipose tissue is a rich source of mesenchymal stem cells (MSCs) containing approximately 500 times more MSCs per gram than bone marrow [2,4-6]. MSCs derived from adipose tissue have been shown to be functionally similar to bone marrow derived MSC (BM-MSC) however some differences may exist [7,8]. There has been substantially more research into the properties and therapeutic potential of BM-MSC over adipose-derived MSCs (ADSC). However, the relative abundance of MSCs in adipose tissue compared to bone marrow, the relative ease of obtaining large volumes of tissue and the ability to rapidly isolate the SVF, makes adipose tissue an attractive source of MSCs.

Historically, the focus on MSCs has been defining their ability to differentiate into cells of the mesenchymal lineage and their subsequent potential therapeutic use. In recent years there has been a shift in focus towards understanding the therapeutic potential of the secretions from MSCs and the SVF. The bioactive factors produced by MSCs and SVF not only modulate the immune system [9-15] but also have angiogenic, anti-apoptotic and anti-scarring properties [see [16], and references therein]. MSCs can modulate the environment and stimulate tissue repair and regeneration. It is clear that MSCs have significant therapeutic potential, either in mixed cell populations or as homogeneous cultured populations. In a number of major jurisdictions, including North America, the European Community and Australia, cell therapy is proceeding along two separate paths, defined by the regulatory environment. In one path, cells that are harvested and then expanded in culture or manipulated in other ways are regulated and only approved for use after extensive safety and efficacy trials. In an alternative approach, a so-called medical exemption exists for treatment in a clinic or hospital under the exclusive professional responsibility of a registered medical practitioner, in order to comply with an individual medical prescription for a custom-made product for an individual patient [17]. This is usually interpreted as encompassing the use of freshly isolated tissue that is minimally manipulated, in-clinic during the same procedure, by enzymatic or mechanical means to obtain a cell population, which is used for autologous treatment. Any attempt to isolate specific cell populations, for example, using antibody coated magnetic beads, would constitute more than minimal manipulation. The use of freshly isolated adipose cell populations such as SVF, alone or in combination with adipocytes, fits within these regulatory frameworks. As adipose tissue can be rapidly processed to prepare a minimally manipulated and un-cultured mixed cell population for therapeutic purposes, its use in cell therapy is increasing. In fact, adipose tissue is unique for this type of cell therapy because of the high frequency of MSCs compared to other tissue sources. There is increasing research, clinical trial and commercial activity along both of the pathways described. The use of cultured MSCs, and uncultured freshly isolated adipose SVF, alone or in combination with adipocytes, are currently available cellular therapies in Australia, Europe and North America.

*In vivo*, the multiple cell types that comprise adipose tissue live in close proximity to each other. Consequently, as part of normal tissue function, there is significant cross-talk between the cells, which occurs though direct cell-cell contact or surface molecular receptors and secreted factors. Indeed, co-culture experiments have demonstrated complex reciprocal signaling, between adipose-derived cells [18-20]. We hypothesized that distinct secretion profiles would be obtained from the *in vitro* culture of various mixed adipose-derived cell populations. To investigate this, we used the largest single multi-plex panel (27 analytes) available for measuring human cytokines, growth factors and chemokines at the protein level. This panel gives broad coverage of inflammatory and anti-inflammatory cytokines, chemokines and growth factors. These analytes were measured in the conditioned medium of 1. the entire cellular fraction of adipose tissue, the SVF co-cultured with adipocytes, 2. SVF alone, 3. adipocytes and 4. adherent MSCs at passage two. A key aspect of this study was to observe the extent to which the secretion profiles obtained from different cell populations were influenced by cross-talk. We chose to address that by comparing the secretion profile of SVF cultured with adipocytes to the profile obtained from adding together the secretion profile of SVF alone with the secretion profile values of adipocytes alone. We refer to this computer-generated secretion profile as an ‘*in silico*’ dataset.

## Methods

### Isolation of human adipose-derived cell populations

This research was approved by the Macquarie University human research ethics committee (Ref #: 5201100385). Written consent was obtained from the patients who participated in this study. Human abdominal lipoaspirates were obtained from five patients undergoing routine liposuction procedures for cosmetic reasons. For each sample, 200 grams of lipoaspirate was digested with 0.5 mg/mL collagenase (Lomb Scientific, USA) mixed with 0.05 mg/mL of vancomycin (Hospira Australia Pty Ltd, Australia) in a 37°C water bath for 30 mins with periodic mixing. The digested samples were passed through an 800 μm mesh and centrifuged at 1500 x *g* for 5 mins to obtain the pelleted cells (SVF) and floating adipocytes. The adipocyte and SVF fractions were washed separately with saline and centrifuged at 1500 x *g* for 5mins. These freshly isolated fractions were placed into culture to produce conditioned medium (see below).

To obtain a population of adherent ADSCs, a portion of each SVF pellet obtained was placed into a T175cm^2^ flask containing Standard Media that consisted of Dulbeccos Modified Eagle Medium (DMEM; Invitrogen, USA) supplemented with 10% foetal bovine serum (FBS; Bovogen, Australia) and 1% Penicillin-Streptomycin solution (Invitrogen, USA). Media changes were performed every 3 days. The initial media change resulted in removal of non-adherent cells. Once the adherent ADSCs reached 80% confluency, cells were passaged using TrypLE express (Invitrogen, USA). Adherent ADSCs were used at passage 2 for the experiments described in this manuscript.

### Confirmation of adherent MSCs

#### Differentiation potential

Adherent ADSCs were obtained from human lipoaspirate samples as described above. ADSCs were seeded at a density of 1 x 10^4^ and 5 x 10^3^ cells per cm^2^ for adipogenic and osteogenic differentiation respectively. Control cells were maintained in Standard Media. Defined adipogenic and osteogenic differentiation media formulations were used as previously described [21]. Media was changed on both control and differentiated wells every 3 days for 3 weeks. Upon completion of differentiation, cells were washed twice with phosphate buffered saline (PBS) and incubated for 30 mins with 4% paraformaldehyde. For adipogenic differentiation, the cells were subsequently washed with MilliQ water, incubated with 60% isopropanol, stained with 0.2% Oil Red O solution for 5 mins at room temperature and washed with tap water. For osteogenic differentiation, the cells were stained with 2% Alizarin red solution for 2 mins at room temperature and washed 3 times with MilliQ water. Control and differentiated cells were imaged using a Carl Zeiss Primo Vert inverted microscope.

#### CD Marker characterization

Adherent ADSCs were obtained from human lipoaspirate samples as described above. Cells were liberated from the flask using TrypLE express, diluted in Standard Media and centrifuged at 2000 x *g* for 5 mins. The cells were washed in PBS and resuspended in PBS with 2% FBS. The cells were stained with the following antibodies, which were all sourced from Becton Dickinson: CD34-FITC (#555821), CD45-FITC (#555482), CD73-PE (#550257), CD90-FITC (#555595), and CD105-PE (#560839) and incubated on ice for 45 mins. Cells were washed with ice cold PBS, centrifuged at 300 x *g* for 5 mins and resuspended in 1x FACS Lysing Solution (Becton Dickinson, USA). The cells stained with FITC conjugated antibodies were resuspended in Propidium iodide (10 μg/mL) and isoflow. Stained and unstained control cells were analysed using a FacsScan flow cytometer (Becton Dickinson, USA).

### Production of conditioned medium

A portion of each of the freshly isolated SVF pellets or cultured adherent ADSCs were filtered through 35 μm nylon mesh topped tubes (Becton Dickinson, USA). Samples were enumerated and the viability determined in TruCount tubes (Becton Dickinson, USA) containing isoflow (Becman Coulter, USA) Propidium iodide (10 μg/mL; Sigma, USA) and Syto11 (1 μM; Invitrogen, USA) using a FacsScan flow cytometer (Becton Dickinson, USA). The total number of viable cells in each of the freshly isolated SVF preparations and adherent cell cultures were determined. On the basis of the viable cell counts, the volume of the freshly isolated SVF or adherent cell suspensions required to seed 1.5 million viable nucleated cells was transferred to separate T75cm^2^ culture flasks. Adipocytes are difficult to accurately enumerate [22] and therefore they were normalised between biological replicates by adding a 3.5 mL volume of the adipocyte enriched fraction. To the co-culture flask, 3.5 mL of freshly isolated adipocytes were added. A volume of 3.5 mL of freshly isolated adipocytes alone was transferred to a T25cm^2^ culture flask. The flask was inverted for incubation to enable optimal contact between the adipocytes and the treated surface of the culture flask. This type of culture is standard for adipocytes and is referred to as ‘ceiling culture’ [23]. The cell suspensions were incubated in Standard Media. All flasks were incubated at 37°C with 5% CO_2_ for 72 hours without media changes. The conditioned medium was collected, centrifuged at 4980 x *g* for 10 mins and stored at −80°C.

### Bio-Plex analysis

Conditioned medium samples were filtered through 0.2 μm Nanosep MF Centrifugal Devices with Bio-Inert® Membrane (Pall Scientific, USA) for 5 mins at 9000 x *g*. Samples (50 μL) of each filtered sample was analysed using the Bio-Plex Pro Human Cytokine 27-plex assay (Bio-Rad, USA) according to the manufacturer’s instructions. The washing steps were performed on the Bio-Plex Pro II magnetic wash station and the data was acquired using the Bio-Plex 200 system with version 5.0 software (Bio-Rad, USA). The resultant cytokine concentrations obtained from the adipocyte ceiling cultures were adjusted for the difference in media volumes. IL-5 has not been included in the data analysis as it was not detected in any of the conditioned medium samples. The average concentration of each cytokine produced by each adipose-derived cell population was calculated from the five biological replicates. To produce the ‘*in silico*’ dataset, the concentration of each cytokine obtained from the *in vitro* culturing of the SVF and adipocytes separately were combined for each biological replicate. The average concentration of each cytokine in this ‘*in silico*’ dataset was then calculated from the five biological replicates. The results are presented as the mean ± SEM. A two-tailed t-test was used to test the significance between the average concentration of each cytokine in the SVF, adipocytes, ADSCs and ‘*in silico*’ datasets compared to the co-culture dataset. Unless otherwise stated, p-values < 0.05 were considered statistically significant.

### Sample preparation and proteomic analysis using iTRAQ

A lipoaspirate sample was processed to obtain the SVF and adipocytes as described above. Four flasks were each seeded with approximately 29 million viable SVF cells. The two flasks designated as the co-culture flasks each received 30 mL of adipocytes and the two SVF only flasks received 30 mL of wash solution (saline with 0.05 mg/mL vancomycin). The volume in each flask was normalized to 80 mL with Standard Media. The flasks were incubated at 37°C with 5% CO_2_ for 72 hours without media changes. Non-adherent cells from each flask were collected from the conditioned medium by centrifugation at 1500 x *g* for 5 mins. Adherent cells were washed in HG DMEM, stripped from each flask using TrypLE express and centrifuged at 1500 x *g* for 5 mins. The non-adherent and adherent SVF cell pellets from each flask were washed in PBS, snap frozen in liquid nitrogen and stored at −80°C. Complete cell lysis and protein denaturation was achieved by adding 1% SDS and 100 mM TEAB to the thawed pellets followed by sonication. At this point, the protein solutions from the adherent and non-adherent cell pellets for each flask were combined. The samples were boiled, sonicated and centrifuged at 21 000 x *g* for 10 mins. Proteins were acetone precipitated, centrifuged at 4980 x *g* for 6 mins and the resultant pellets were solubilized in 1% SDS and 100mM TEAB followed by sonication, boiling and centrifugation at 21 000 x *g* for 5 mins. Samples were processed through columns (micro Bio-spin, P6 in SSC; Bio-RAD, USA) according to the manufacturer’s instructions. 100 μg of each of the 4 samples was reduced with tris(2-carboxyethyl)phosphine, alkylated with methyl methanethiosulfonate and digested with trypsin. The resultant peptides from each sample were labeled with 1 of the 4 iTRAQ labels (114, 115, 116 or 117) according to the manufacturer’s instructions (AB Sciex, USA).

The labeled samples were cleaned and fractionated by SCX HPLC. Buffer A was 5 mM phosphate and 25% acetonitrile with a pH of 2.7 and buffer B was 5 mM phosphate, 350 mM potassium chloride and 25% Acetonitrile with a pH 2.7. The dried iTRAQ labeled samples were resuspended in buffer A. After sample loading and washing with buffer A, buffer B concentration was increased from 10% to 45% in 70 mins, increased quickly to 100% and maintained at 100% for 10 mins at a flow rate of 300 μl/min. The eluent from SCX was collected every 2 mins at the beginning of the gradient and at 4 minutes intervals thereafter. The SCX fractions were each concentrated and desalted using a Michrome peptide Captrap (Michrom Bioresources, USA). The peptide trap was then switched into line with the 150 μm x 10 cm C18 3 μm 300 A ProteCol analytical column (SGE, Australia). Peptides were eluted from the column using a 120 min linear solvent gradient. The reverse phase nanoLC eluent was subject to positive ion nanoflow electrospray analysis in an information dependant acquisition mode (IDA) on a Qstar Elite mass spectrometer (AB Sciex, USA). In IDA mode a TOFMS survey scan was acquired (m/z 370–1600, 0.5 second), with the three most intense multiply charged ions (counts >70) in the survey scan sequentially subjected to MS/MS analysis. MS/MS spectra were acquired in the mass range m/z 100–1600. The experimental nanoLC ESI ms/ms data were submitted to ProteinPilot V4.0 (AB Sciex, USA) for data processing using Homo sapiens species. Bias correction was selected. The detected protein threshold (unused ProtScore) was set as larger than 1.3 (better than 95% confidence). FDR (False discovery rate) analysis was selected.

## Results

### Confirmation of MSCs

A cell is recognized as an MSC if it meets the criteria outlined in the International Society for Cellular Therapy position statement, which involves displaying plastic adherence, the ability to differentiate into specialized mesenchymal cells, expression of CD73, CD90 and CD105 and lack of expression of the hematopoietic markers, CD34 and CD45 [24]. To confirm the adherent cell population obtained from the SVF in this study were MSCs, we analyzed the differentiation capabilities and CD marker profiles of these cells. Following treatment with standard adipogenic and osteogenic differentiation media formulations, the adherent cells stained positive for lipid accumulation following Oil Red O staining (Additional file 1: Figure S1a) and for calcium deposition with Alizarin red staining (Additional file 1: Figure S1c). Control cells were negative for lipid accumulation and calcium deposition (Additional file 1: Figure S1b and d respectively). The CD marker characterization demonstrated that the adherent cells expressed CD73, CD90 and CD105, but lacked expression of CD34 and CD45 (data not shown). These results confirm that the adherent cells obtained from the SVF meet the MSC identification criteria.

### Analysis of overall cytokine secretion profiles from adipose derived cell populations

To compare the effect of co-culturing the entire cellular fraction (the co-culture dataset) isolated from adipose tissue, versus the SVF and adipocyte fractions separately, an ‘*in silico*’ dataset was produced. This was achieved by adding the individual secretion profile values obtained from the two separate fractions. This ‘*in silico*’ dataset has been included in all analyses.

**Figure 1:**
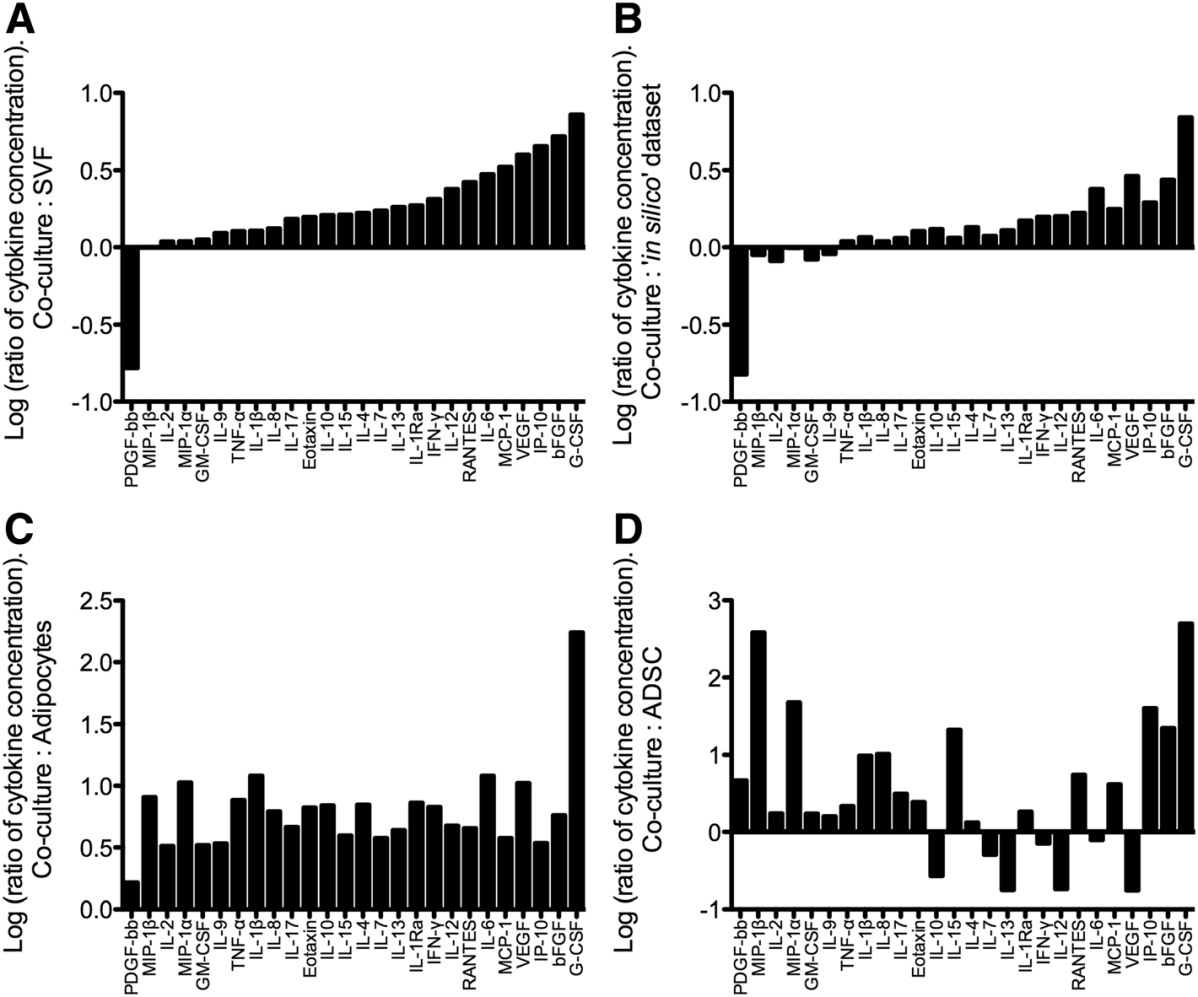
**Comparison of secretion profiles of the SVF co-cultured with adipocytes compared to the SVF, adipocytes, ADSCs and ‘*in silico*’ datasets.** The log of the ratio of each cytokine concentration between the stromal vascular fraction (SVF) co-cultured with adipocyte results (co-culture) and the SVF (**A**), ‘*in silico*’ dataset (**B**), adipocytes (**C**) and the adipose-derived stem cells (ADSCs; **D**) was calculated. The cytokines are ordered based on the comparison between the co-culture and SVF datasets (**A**).

As the co-culture samples contained the entire cellular fraction of adipose tissue, this dataset was used as the reference to examine the differences in the secretion profiles obtained from the other adipose-derived cell populations. A log of the ratio of individual cytokine concentrations in the co-culture dataset compared to the other cell populations studied and the ‘*in silico*’ dataset was calculated. The results are illustrated in Figure1 in which the cytokines are presented in the same order in all graphs. From Figure1A, it is evident that with the exception of PDGF and MIP-1β, increased levels of the cytokines measured were secreted from the co-culture samples when compared to the SVF alone. A similar trend was seen when the co-culture samples were compared to the ‘*in silico*’ dataset (Figure1B). However, a number of the cytokines in this comparison were present at similar levels in the conditioned medium from the two sample types. The levels of all 26 cytokines were present in higher concentrations in the co-culture dataset when compared to the adipocyte only dataset, with G-CSF exhibiting the highest increase of 14,242 pg/mL (174-fold increase; Figure1C). The comparison between the co-culture and ADSC datasets exhibited the greatest fluctuations in cytokine concentrations (Figure1D). There were 6 cytokines (IL-7, IL-10, IL-12, IL-13, IFN-γ and VEGF) secreted at higher concentrations by ADSCs, and 2 (IL-2, and IL-6) that were secreted in similar concentrations by both sample types. The remaining 18 cytokines were secreted at higher concentrations by the co-culture samples.

To investigate whether any trends were evident in the cytokines secreted in higher or lower levels by each adipose-derived cell population, the cytokines were categorized into pro-inflammatory, anti-inflammatory, chemokines, growth factors and those with dual roles. Following categorization, a two-tailed t-test was performed to determine if the concentration of each cytokine secreted by the SVF, adipocytes, ADSCs, and the ‘*in silico*’ datasets were statistically different from the co-culture dataset.

### Pro-inflammatory cytokines

**Figure 2:**
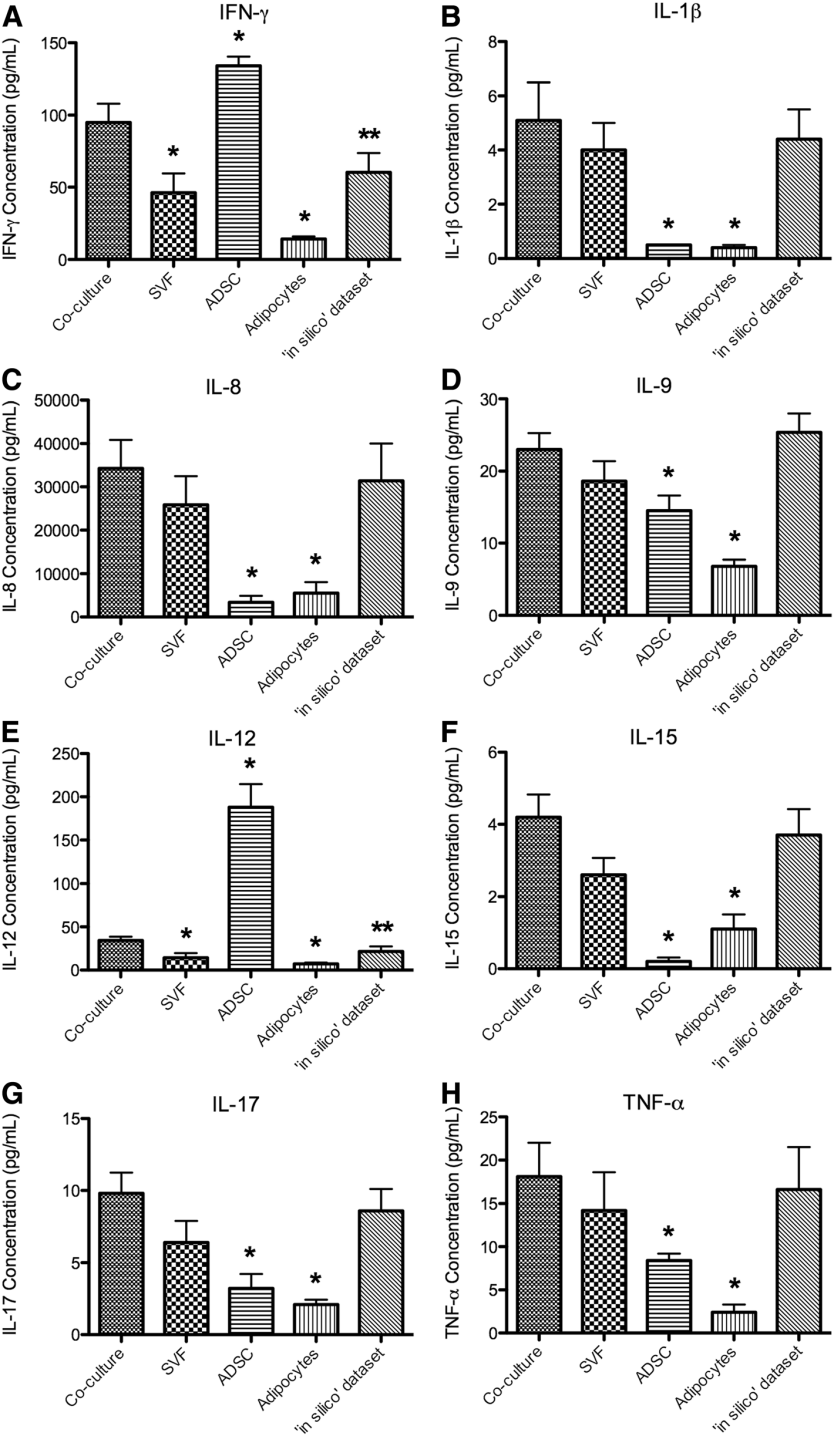
**Average concentration of pro-inflammatory cytokines secreted by adipose-derived cell populations.** The concentrations of the pro-inflammatory cytokines, IFN-γ (**A**), IL-1β (**B**), IL-8 (**C**), IL-9 (**D**), IL-12 (**E**), IL-15 (**F**), IL-17 (**G**) and TNF-α (**H**) were measured in the conditioned medium of the stromal vascular fraction (SVF) co-cultured with adipocytes (co-culture), SVF, adipose-derived stem cells (ADSC), and adipocytes, as well as the ‘*in silico*’ dataset. The reported values are the average of 5 biological replicate ± SEM. An * denotes a p-value of ≤ 0.05 and an ** denotes a p-value ≤ 0.1 when compared to the co-culture dataset.

Of the 27 cytokines measured in this study, 8 are considered to be pro-inflammatory based on their *in vivo* and *in vitro* activities defined in the literature. IFN-γ and IL-12 were significantly higher in the co-culture dataset when compared to the SVF and adipocyte samples (p-value < 0.05) and the ‘*in silico*’ dataset (p-value < 0.1; Figures 2A and E). In contrast, ADSCs produced significantly higher levels of these two cytokines when compared to the co-culture dataset (p-value < 0.05; Figures 2A and E). The levels of the remaining pro-inflammatory cytokines were significantly higher in the co-culture dataset when compared to the ADSC and adipocyte datasets (p-value < 0.05; Figure2).

### Anti-inflammatory cytokines

**Figure 3:**
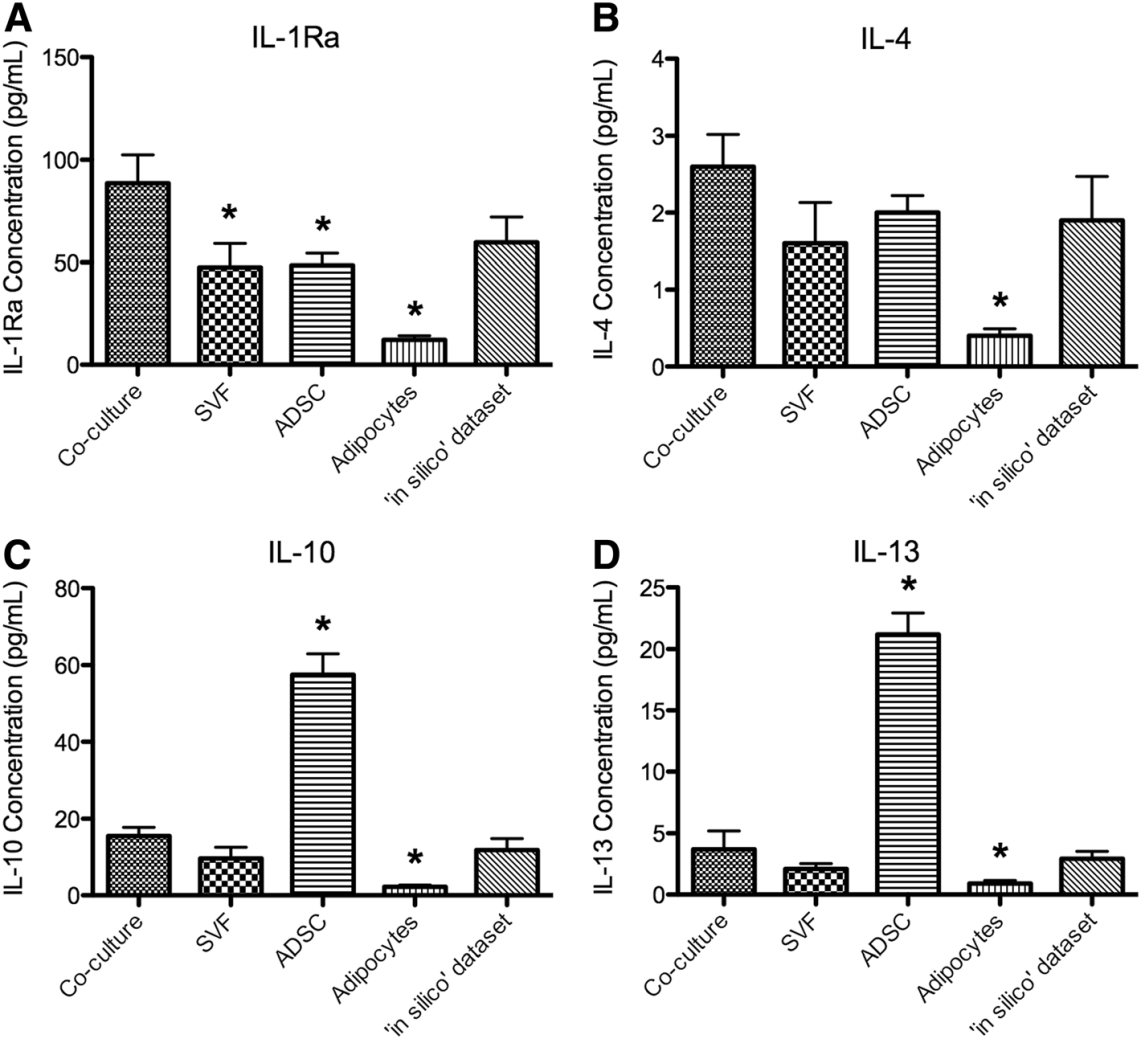
**Average concentration of anti-inflammatory cytokines secreted by adipose-derived cell populations.** The concentrations of the anti-inflammatory cytokines, IL-1Ra (**A**), IL-4 (**B**), IL-10 (**C**), IL-13 (**D**), were measured in the conditioned medium of the stromal vascular fraction (SVF) co-cultured with adipocytes (co-culture), SVF, adipose-derived stem cells (ADSC), and adipocytes, as well as the ‘*in silico*’ dataset. The reported values are the average of 5 biological replicate ± SEM. An * denotes a p-value of ≤ 0.05 when compared to the co-culture dataset.

A comparison of the ‘*in silico*’ and co-culture datasets showed no statistical differences between the levels of the anti-inflammatory cytokines, IL-1Ra, IL-4, IL-10 and IL-13 measured. The co-culture dataset had significantly higher levels of IL-1Ra than the other cell populations studied (p-value < 0.05) but was not significantly different from the ‘*in silico*’ dataset (Figure3A). In contrast, IL-4 was produced in significantly lower concentrations by the adipocyte fraction only when compared to the co-culture dataset (p-value = 0.001; Figure3B). IL-10 and IL-13 were produced in significantly higher levels by ADSCs and significantly lower levels by adipocytes when compared to the co-culture dataset (p-values < 0.05; Figures 3C and D).

### Chemokines

**Figure 4:**
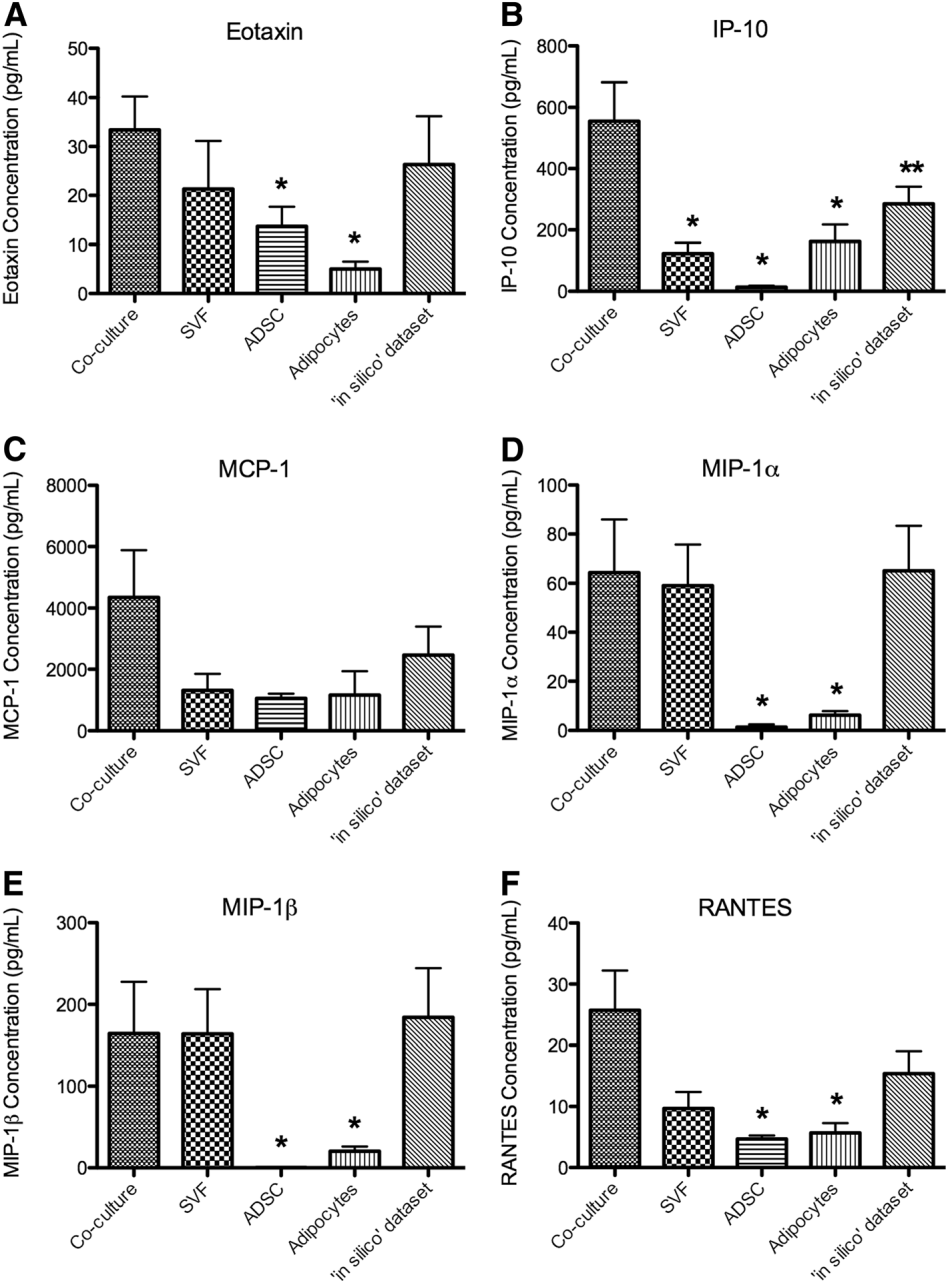
**Levels of chemokines measured in conditioned medium of adipose-derived cell populations.** The concentrations of the chemokines, eotaxin (**A**), IP-10 (**B**), MCP-1 (**C**), MIP-1α (**D**), MIP-1β (**E**) and RANTES (**F**) were measured in the conditioned medium of the stromal vascular fraction (SVF) co-cultured with adipocytes, SVF, adipose-derived stem cells (ADSC), and adipocytes, as well as the ‘*in silico*’ dataset. The reported values are the average of 5 biological replicate ± SEM. An * denotes a p-value of ≤ 0.05 and an ** denotes a p-value ≤ 0.1 when compared to the co-culture dataset.

Eotaxin, MIP-1α, MIP-1β and RANTES all exhibited similar profiles with significantly lower concentrations produced by the ADSC and adipocyte samples when compared to the co-culture dataset (p-value < 0.05; Figures 4A, D, E and F respectively). The co-culture dataset contained significantly higher concentrations of IP-10 than the other cell populations studied (p-value < 0.05) and the ‘*in silico*’ dataset (p-value < 0.1; Figure4B). There were no significant differences between the levels of MCP-1 in the co-culture dataset and the other cell populations studied (Figure4C).

### Growth factors

**Figure 5:**
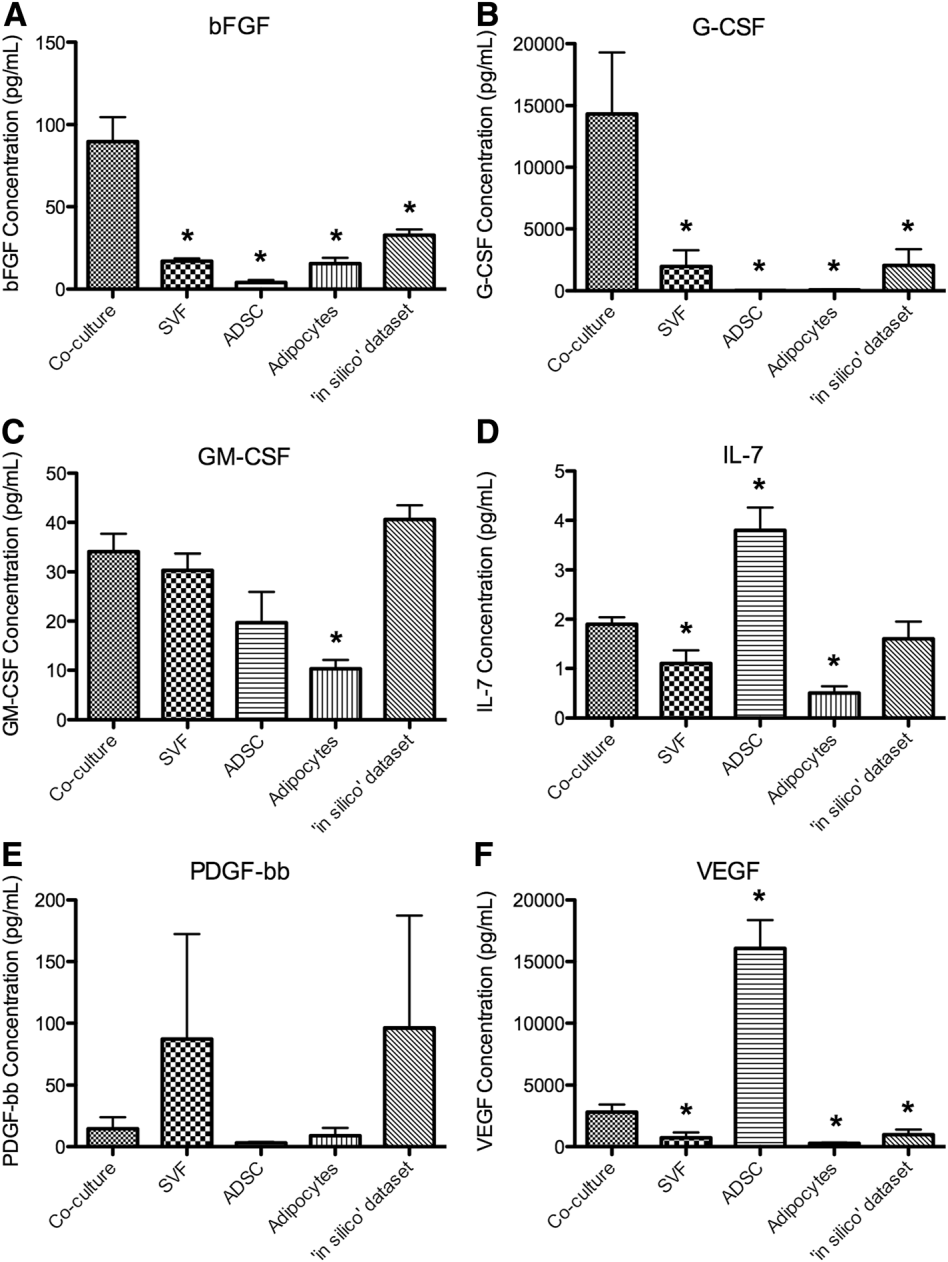
**Concentration of growth factors measured in conditioned medium of adipose-derived cell populations.** The concentrations of the growth factors, bFGF (**A**), G-CSF (**B**), GM-CSF (**C**), IL-7 (**D**), PDGF-bb (**E**) and VEGF (**F**) were measured in the conditioned medium of the stromal vascular fraction (SVF) co-cultured with adipocytes, SVF, adipose-derived stem cells (ADSC), and adipocytes, as well as the ‘*in silico*’ dataset. The reported values are the average of 5 biological replicate ± SEM. An * denotes a p-value of ≤ 0.05 when compared to the co-culture dataset.

bFGF and G-CSF exhibited similar profiles with significantly lower concentrations seen in all cellular fractions studied (including the ‘*in silico*’ dataset) when compared to the co-culture dataset (p-values < 0.05; Figure5A and B). The co-culture dataset had significantly higher concentrations of GM-CSF than the adipocyte only dataset (p-value = 4.2E-4; Figure5C). The conditioned medium from ADSCs contained significantly increased concentrations of IL-7 and VEGF than the co-culture dataset (p-values = 0.004 and 0.001 respectively; Figures 5D and F). SVF and adipocytes however produced significantly lower levels of IL-7 and VEGF than the co-culture dataset (p-value < 0.05; Figures 5D and F). Furthermore, VEGF levels were significantly lower in the ‘*in silico*’ dataset when compared to the co-culture samples (p-value = 0.038; Figure5F). Interestingly, large biological variation was seen in the levels of PDGF in the SVF samples. The secreted concentration of PDGF was not significantly different in the ‘*in silico*’ dataset or any of the cell populations analysed when compared to the co-culture dataset (Figure5E).

### Cytokines with dual roles

**Figure 6:**
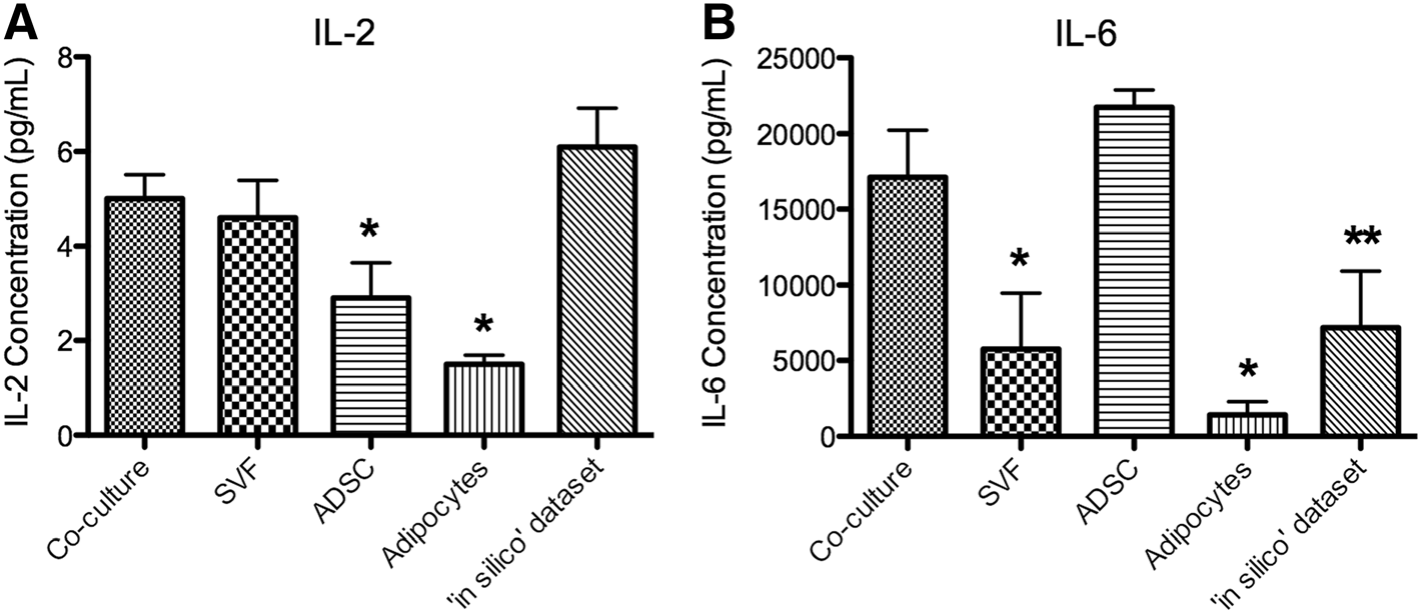
**Concentration of cytokines with dual roles measured in conditioned medium of adipose-derived cell populations.** The concentrations of the cytokines, IL-2 (**A**), and IL-6 (**B**) were measured in the conditioned medium of the stromal vascular fraction (SVF) co-cultured with adipocytes, SVF, adipose-derived stem cells (ADSC), and adipocytes, as well as the ‘*in silico*’ dataset. The reported values are the average of 5 biological replicate ± SEM. An * denotes a p-value of ≤ 0.05 and an ** denotes a p-value ≤ 0.1 when compared to the co-culture dataset.

IL-2 and IL-6 are the 2 cytokines in the Bio-Plex panel considered to have both pro- and anti-inflammatory activities under different conditions. The ADSC and adipocyte samples secreted significantly lower levels of IL-2 when compared to the co-culture dataset (p-values = 0.049 and 2.3E-4 respectively; Figure6A). IL-6 was secreted in significantly lower levels by both the SVF and adipocyte fractions (p-value < 0.05) as well as the ‘*in silico*’ dataset (p-value < 0.1) when compared to the co-culture dataset (Figure6B).

### Summary of the Bio-Plex data

Table1 contains a summary of the number of statistically significant cytokines between each of the fractions (SVF, adipocytes, ADSCs and ‘*in silico*’ datasets) with respect to the co-culture dataset, which contained secretions from the entire cellular makeup of adipose tissue. The *in vitro* co-culture of the SVF with adipocytes resulted in significantly increased secretion of 9, 15, 23 and 3 factors when compared to the SVF, ADSC, adipocyte and ‘*in silico*’ datasets respectively (p-value < 0.05). However, an additional 4 factors were present in significantly increased concentrations (p-value < 0.1) in the co-culture datasets when compared to the levels seen in the ‘*in silico*’ dataset. There were no cytokines secreted in significantly higher concentrations by the SVF alone, adipocytes alone, or the calculated ‘*in silico*’ dataset when compared to the co-culture dataset. However, isolation and subsequent culturing of adherent ADSCs did result in 6 cytokines being secreted in significantly higher concentrations than in the co-culture dataset.

**Table 1:** **Summary of *in vitro *secretion profiles from adipose-derived cell populations**

	**Number of cytokines significantly decreased**^**a**^	**Number of cytokines significantly increased**^**a**^
SVF	9	-
ADSC	15	6
Adipocytes	23	-
'*in silico*' dataset	3	-

^a^ Number of cytokines produced in significantly (p-value < 0.05) increased or decreased levels by the different adipose-derived cell populations tested when compared to the co-culture dataset.

### iTRAQ proteomic analysis of the SVF intra-cellular proteome cultured in the presence or absence of adipocytes

The *in vitro* co-culture of the SVF with adipocytes resulted in a distinct secretion profile when compared to the secretion profiles obtained from the SVF alone, adipocytes alone and the ‘*in silico*’ dataset. To supplement the secretion profile data, we wanted to determine if co-culture or isolated culture would have any significant effects on the intra-cellular proteome of the SVF. We were particularly interested in whether co-culture or isolated culture would affect the cells in a negative way that may, in turn, affect their suitability as a therapeutic. To achieve this we used a quantitative proteomic approach, iTRAQ, to examine the intra-cellular proteome of the SVF following a 72-hour culture in the presence or absence of adipocytes. The 4-plex iTRAQ system enables a comparison of 4 different samples by labeling the tryptic peptides from each sample with a different isobaric tag. Following fragmentation by collision-induced dissociation, reporter ions of different masses are generated and the ratios of these reporter ion peak areas are subsequently used to determine the relative abundance of the peptides from the 4 samples. In our experimental design, we chose to analyze technical replicates of the SVF intra-cellular proteome following culture in the presence or absence of adipocytes in a 4-plex iTRAQ system. We identified a total of 2200 proteins from 23,073 distinct peptides, which were identified from 70475 spectra using this method. In each comparison between the SVF intra-cellular proteome incubated with or without adipocytes, a threshold of > 1.2 and < 0.8 was assigned for the iTRAQ ratio intensities obtained. This represents an increase or decrease in protein concentration of more than 20%. Table2 contains the protein identifications, percentage of protein coverage, number of unique peptides, and fold change of the protein in each comparison of the SVF proteome incubated with adipocytes versus the SVF alone for all 24 proteins that showed a significant change (p < 0.05). From this table, it is evident that of the 24 proteins which were significantly changed, 17 were up-regulated and 7 were down-regulated in the intra-cellular proteome of the SVF incubated with adipocytes when compared to the SVF incubated alone. It is evident from the differentially expressed proteins in Table2 that short-term *in vitro* culture of the SVF in the absence of adipocytes did not result in massive changes in the intra-cellular proteome of the SVF. This result indicates that under these experimental conditions the absence or presence of adipocytes did not appear to activate stress responses in the SVF that caused substantial changes at the intra-cellular proteomic level.

**Table 2:** SVF intra-cellular proteome differences following culturing in the presence or absence of adipocytes

**UniProt accession No.**	**Protein Name**	**% Coverage (No. of peptides)**	**SVF (+) : SVF (−) 115/116**	**SVF (+) : SVF (−) 114/117**
**Significant Up-Regulated Proteins**				
O60240	Perilipin-1	31 % (9)	2.73	2.44
P33121	Long-chain-fatty-acid--CoA ligase 1	19 % (10)	1.71	2.01
Q99541	Perilipin-2	46 % (15)	1.65	1.34
P23141	Liver carboxylesterase 1	42 % (20)	1.63	1.50
Q9BRX8	Uncharacterized protein C10orf58	31 % (8)	1.53	
Q16853	Membrane primary amine oxidase	51 % (52)	1.51	1.35
P21695	Glycerol-3-phosphate dehydrogenase [NAD+], cytoplasmic	24 % (5)	1.46	1.52
Q12797	Aspartyl/asparaginyl beta-hydroxylase	31 % (17)	1.38	1.39
P05091	Aldehyde dehydrogenase, mitochondrial	43 % (19)	1.37	1.37
Q14315	Filamin-C	28 % (37)	1.29	1.56
Q9BXN1	Asporin	33 % (8)	1.29	1.29
Q16836	Hydroxyacyl-coenzyme A dehydrogenase, mitochondrial	39 % (9)	1.29	
Q9UHG3	Prenylcysteine oxidase 1	30 % (11)	1.28	
P49327	Fatty acid synthase	20 % (29)	1.17	1.48
P42765	3-ketoacyl-CoA thiolase, mitochondrial	50 % (12)	1.17	1.29
P05120	Plasminogen activator inhibitor 2	57 % (24)	1.13	1.50
P09601	Heme oxygenase 1	53 % (15)	1.13	1.41
P15090	Fatty acid-binding protein, adipocyte	85 % (24)	0.86	0.54
P30740	Leukocyte elastase inhibitor	28 % (8)	0.81	0.70
P04179	Superoxide dismutase [Mn], mitochondrial	50 % (14)	0.77	
P29966	Myristoylated alanine-rich C-kinase substrate	42 % (9)	0.73	
P43490	Nicotinamide phosphoribosyltransferase	45 % (15)	0.68	0.66
P40261	Nicotinamide N-methyltransferase	22 % (4)	0.60	0.65
P20591	Interferon-induced GTP-binding protein Mx1	22 % (8)	0.59	

Replicate SVF samples were cultured in the presence (+) or absence (−) of adipocytes for 72 hours in Standard Media. The SVF cells were collected, prepared and the tryptic peptides were labeled with an isobaric tag (114, 115, 116 or 117) for iTRAQ analysis using a Qstar Elite. A total of 2220 proteins were identified but only those which were significantly (p-value < 0.05) differentially expressed between the proteomes of the SVF cultured in the presence (+) or absence (−) of adipocytes have been included. For each of these proteins, the UniProt identification number, protein name, percentage of protein coverage (and number of unique peptides contributing to the sequence coverage), and fold change between the proteins in the SVF cultured with adipocytes versus the SVF cultured alone, for both replicates, are included.

## Discussion

The purpose of this study was to compare the secretion profiles obtained from human adipose-derived cell populations. We used a panel of 27 cytokines to analyse the *in vitro* secretion profiles obtained from the entire cellular composition of adipose tissue (the co-culture dataset), SVF alone, adipocytes alone and cultured ADSCs. Furthermore, we produced an ‘*in silico*’ dataset by combining the individual secretion profiles obtained from culturing the SVF with that of the adipocytes. This ‘*in silico*’ dataset was used to determine what effect, if any, the cross-talk between SVF and adipocytes, had on the secretion profiles. We found that co-culturing the SVF with adipocytes resulted in significantly increased concentrations of between 3 and 23 cytokines when compared to the other cell populations studied (Table1). Interestingly our results illustrate that no component of adipose tissue secretes an exclusive anti-inflammatory or pro-inflammatory profile. Instead, the cytokine secretion profiles vary between each of the 4 cellular fractions of adipose tissue as well as the ‘*in silico*’ dataset. We also used quantitative proteomics (iTRAQ) to determine if co-culture or isolated culture would have any significant effects on the intra-cellular proteome of the SVF. We found 17 proteins significantly up-regulated and 7 proteins significantly down-regulated in the proteome of the SVF co-cultured with adipocytes when compared to the proteome of the SVF cultured alone. However, none of these proteins indicated that the presence or absence of adipocytes under these experimental conditions was inducing stress-responses in the SVF cells resulting in negative changes in the cellular proteome of these cells.

### Increased secretion of cytokines by the co-culture of SVF with adipocytes

The ‘*in silico*’ dataset of SVF and adipocytes produces a similar secretion profile to the co-culture of SVF and adipocytes. However, co-culturing the SVF with adipocytes produced significantly higher concentrations of 3 cytokines (p < 0.05) and an additional 4 with a p < 0.1 than the ‘*in silico*’ dataset. In particular, the co-culture of SVF with adipocytes produced a growth factor boost with VEGF, G-CSF and bFGF increased by 2.89, 6.97 and 2.74 fold respectively compared to the ‘*in silico*’ dataset. This result suggests that the SVF and adipocyte cells are interacting in a manner that enhances their paracrine signaling by inducing the production of these factors by one or more of the cell types. These secretion data support that hypothesis and demonstrate that analyzing individual cell populations will provide an incomplete understanding of the cytokine activity of adipose tissue. The analysis of the secretion profiles of these cell populations is one way to illustrate their paracrine signaling activities. However, cells can also use mRNA and microRNA in microvesicles to communicate [25], although the downstream effects on protein expression and secretion profiles is not clear. It is emerging that there may be a variety of non-protein cross talk mechanisms between cells that were not investigated in this study. Our objective was not a comprehensive analysis of the cross-talk in adipose tissue, rather to use secretion profiling and proteomics to understand the likely *in vivo* potency of mixed cell vs homogeneous populations.

Considering the SVF cells reside in close proximity to adipocytes *in vivo*, and adipose tissue plays a role in a number of processes within the body, substantial cross-talk between the cells in adipose tissue is not unexpected. Indeed, *in vitro* experiments have shown significant cross-talk between adipocytes and other cells contained within the SVF such as lymphocytes and macrophages [19,20,26]. Adipocytes are therefore not passive storage cells but instead actively secrete a wide variety of hormones, growth factors, chemokines and cytokines. Interestingly, no cytokines measured in this study were found to be significantly increased in the ‘*in silico*’ dataset, possibly indicating that positive feedback mechanisms are involved when adipocytes were co-cultured with the SVF.

In this study, the *in vitro* culture of the SVF alone yielded decreased concentrations of all cytokines measured, compared to the cytokine profile of co-cultured adipocytes and SVF. Out of the 26 cytokines, 9 were significantly lower (p < 0.05) in the SVF, when compared to the co-culture samples. As no cytokines were secreted in significantly higher concentrations by the SVF only samples, it is consistent with our observations that the co-culture of adipocytes with the SVF involves positive feedback mechanisms and does not appear to suppress the production of the cytokines measured in this study.

### Increased secretion of cytokines by ADSCs

The *in vitro* culturing of ADSCs resulted in the secretion of significantly higher concentrations (p < 0.05) of 6 cytokines (IFN-γ, IL-7, IL-10, IL-12, IL-13, and VEGF) when compared to the co-culture dataset. Consistent with standard approaches in the literature [27,28], the ADSC population in this study was isolated from the SVF by selecting for plastic adherence and subsequently expanding the cells to passage two *in vitro.* This is a more selective cell population when compared to the SVF and co-culture samples. Consequently, the increased secretion of some cytokines by the isolated adherent ADSCs, when compared to co-culture samples, may be due to the increase in cell numbers during culture and the lack of immune cells and adipocytes regulating their secretions.

### Quantitative proteomic analysis of the SVF proteome following in vitro culture in the presence or absence of adipocytes

We found 17 proteins significantly up-regulated and 7 proteins significantly down-regulated between the intra-cellular proteome of the SVF from *in vitro* co-culture with adipocytes or isolated culture. Of the 17 proteins significantly up-regulated, there were a number of prominent abundant adipocyte proteins including perilipin-1 [29], perilipin-2 [30], liver carboxyesterase 1 [31], membrane primary amine oxidase [32], glycerol-3-phosphate dehydrogenase [33], and fatty acid synthase [34]. Membrane primary amine oxidase, for example, is a major component of the adipocyte plasma membrane [32]. The most likely explanation for the increased level of these proteins is contamination of the SVF intra-cellular proteome with adipocyte proteins despite our use of density differentials to separate the adipocytes from the SVF and the subsequent stringent washing of the SVF. However, it is also possible that different cell types, including the ADSC population in the SVF that can give rise to adipocytes, may produce these proteins. Lastly, some early ADSC differentiation into adipocytes may be occurring resulting in an increase in the levels of these proteins. Differentiation is unlikely as a source of these proteins because the widely accepted method of *in vitro* ADSC differentiation into adipocytes requires the use of differentiation media and takes at least 2 weeks to complete [21]. Therefore, it is unlikely that considerable differentiation of the ADSCs in the SVF preparation occurred in the short-time frame of 3 days *in vitro* prior to iTRAQ analysis. In addition, the presence of mature adipocytes has been shown *in vitro* to inhibit the differentiation of adherent adipose-derived cells into adipocytes [35].

Of the proteins significantly down-regulated in the proteome of the SVF cells co-cultured with the adipocytes when compared to the isolated culture of the SVF, the identification of fatty acid-binding protein, adipocyte (FABP4) was of interest. It has been reported that increased expression of the gene encoding this fatty acid-binding protein promotes the differentiation of MSCs into adipocytes [36]. Indeed, enhanced expression of the gene encoding FABP4 has been reported from human ADSCs grown on different scaffolds and subjected to adipogenic stimulants [37]. Therefore, it is possible that some of the cells in the SVF cultured alone may have started to differentiate into adipocytes due to the absence of mature adipocytes. This may explain the increased levels of FABP4 measured in this sample. Any differentiation that had initiated in the SVF or co-culture experiments would have been at a preliminary stage when the cells were harvested. An in-depth understanding of differentiation among mixed cell populations *in vitro* would require considerably increased culture time and the results would be different from the *in vivo* situation.

Although a quantitative proteomic analysis of the SVF co-cultured with adipocytes or cultured alone resulted in the differential expression of 24 proteins, the functions of these proteins do not indicate that a stress response is being induced in the SVF under either condition. Instead, it appears that the SVF cells are functioning normally at the intra-cellular proteome level *in vitro* in the presence or absence of adipocytes. A cellular therapeutic of either the SVF supplemented with adipocytes or the SVF alone is likely to be held for only minutes to hours before re-introduction into the patient. Therefore, from this iTRAQ data it appears that the absence or presence of adipocytes is unlikely to have any significant effects on the intra-cellular proteome of the SVF during this holding period. However, the Bio-Plex data indicates that the cellular signaling capacity of the SVF at the cytokine level is being affected by the presence or absence of adipocytes.

### Therapeutic importance

There have been numerous *in vivo* and *in vitro* studies demonstrating the paracrine activities of ADSCs. The use of freshly isolated populations from adipose tissue such as the SVF, alone or in combination with adipocytes, is becoming increasingly common as a cellular therapy. This is not only due to the high frequency of MSCs in adipose tissue, but also because the procedure can be performed in-clinic under a medical exemption, as the procedure is autologous, the tissue is minimally manipulated and it is performed as an out-patient procedure under the responsibility of a registered medical practitioner. Consequently the secretion profiles produced by these isolated and mixed cell populations are of interest in order to delineate functional differences and guide potential therapeutic use. Although this *in vitro* study may not reflect the secretory activities of these cell types in *in vivo* models of disease or real clinical cases, it provides some insight into the secretion capabilities of different adipose-derived cellular fractions. These data also support the clinical results obtained via the use of freshly isolated adipose-derived mixed cell populations [3]. Furthermore, these adipose-derived secretions may also be of importance for future off-the-shelf secretion based therapeutics.

The use of a single bioactive factor for the treatment of a variety of conditions has been trialed in both animals with induced diseases and humans with clinical conditions. Given the pleiotropic nature and multi-factorial roles of most cytokines, the introduction of a single cytokine in an unregulated fashion is likely to produce unwanted effects. Indeed, in a number of studies, adverse reactions were observed [38-40] and references therein]. In some cases, the side effects observed following administration of a single cytokine were considered a direct consequence of inappropriate immune system activation. Furthermore, the administration of multiple bioactive factors has provided an improved beneficial outcome over factors administered individually [41-45].

Cellular therapy may offer advantages over administration of cytokines, as it appears the introduced cells are able to respond to the local environment and as a result, secrete an appropriate mixture of bioactive factors. The relative ease of harvest of adipose tissue, compared to bone marrow, makes it an ideal source of cells for autologous cell therapy. Collagenase digestion of freshly isolated adipose tissue typically produces millions of viable cells per gram of fat, thus enabling day surgery in-clinic procedures using autologous, uncultured mixed cell populations. The use of adipose mixed cell populations such as the SVF with adipocytes may be an option for the treatment of conditions where reduction of inflammation is a key aspect of the treatment. The therapeutic effect is likely to proceed in two phases after *in vivo* implantation. Firstly, the freshly isolated, combined population of SVF and adipocytes has the capacity to secrete high levels of cytokines of therapeutic and immuno-modulatory importance, such as IL-1Ra (Figure3). Following *in vivo* introduction of the SVF with adipocytes, it is possible that high levels of cytokines of therapeutic importance may be produced. The high levels of ADSCs in adipose SVF constitute the second phase, where these cells are likely to embed in the host tissue and continue to control the microenvironment through their secretions including production of the anti-inflammatory cytokines, IL-10 and IL-13 (Figure3). Indeed, ADSCs when introduced systemically can home to the site of injury or inflammation, embed and stimulate regeneration of the tissue, through secretion of bioactive factors that control the microenvironment [46,47]. However, embedding of non-stem cells cannot be discounted when mixed cell populations are introduced. To date, there has been no agreement in the literature regarding a defined number of cells or minimal concentration of cells or cytokines required to achieve a therapeutic outcome. Therefore further *in vivo* work needs to be conducted in various disease models to define at least the lower limits for efficacy.

## Conclusions

In this study we have demonstrated that a short-term *in vitro* co-culture of SVF with adipocytes or isolated culture of SVF does not have any negative effects on the intra-cellular proteome of the SVF that would affect their suitability as therapeutics. However, we illustrated that the *in vitro* culture of various combinations of human adipose-derived cell populations results in significantly different cytokine secretion profiles. In particular, a combined culture of SVF and adipocytes results in a distinct secretion profile when compared to the ‘*in silico*’ dataset, produced by combining the secretion profiles of the isolated SVF with that of isolated adipocytes. This illustrates that co-culture of the SVF with adipocytes results in the cells acting in a synergistic manner and modulating the production of cytokines by one or more cell types. The use of a mixed cell population such as the SVF with adipocytes may be considered for the treatment of conditions which have inflammatory components. The therapeutic effect is likely to proceed in two phases after *in vivo* implantation. Firstly, the freshly isolated, combined population of SVF and adipocytes has the capacity to secrete high levels of cytokines of therapeutic and immuno-modulatory importance, such as IL-1Ra. Achieving a reduction in inflammation in injured or inflamed tissues is required to firstly halt tissue destruction and then subsequently enable tissue repair to occur [48]. The high levels of ADSCs in adipose SVF constitute the second phase, where these cells are likely to embed in the host tissue, continue to control the microenvironment through their paracrine activities and stimulate the surrounding cells to repair and regenerate the damaged tissue [46,47,49].

## Abbreviations

SVF: Stromal vascular fraction; MSC: Mesenchymal stem cell; BM-MSC: Bone marrow-derived mesenchymal stem cell; ADSC: Adipose-derived mesenchymal stem cell; DMEM: Dulbeccos modified eagle medium; FBS: Foetal bovine serum.

## Competing interests

Sinead Blaber, Rebecca Webster and Graham Vesey are employees of Regeneus Ltd. Benjamin Herbert is a consultant to Regeneus Ltd. Regeneus Ltd provided some funding for this research.

## Authors’ contributions

SB was involved in the conception and design of this study, collection and assembly of data, data analysis and interpretation and manuscript writing. RW was involved in collection and assembly of data, data analysis and interpretation and manuscript writing. CH was involved in the collection and assembly of data, data analysis and interpretation and manuscript writing. EB was involved in data analysis and interpretation. DK was involved in manuscript writing. GV was involved in conception and design of this study, providing financial support, data analysis and interpretation and manuscript writing. BH was involved in conception and design of this study, providing financial support, data analysis and interpretation and manuscript writing. All authors read and approved the final manuscript.

## Supplementary Material

Additional file 1 **Figure S1. Differentiation of adherent human adipose-derived cells into adipogenic and osteogenic lineages.** Adherent cells were obtained from human lipoaspirate samples and treated with standard differentiation media or control media. Adipogenic differentiated (A) and control (B) cells were stained with Oil Red O to visualize lipid accumulation. Osteogenic differentiated (C) and control (D) cells were stained with Alizarin Red to visualize calcium deposition. Click here for file
