# Supplementary figures and images for: Analysis of in vitro secretion profiles from adipose-derived cell populations
Source: J Transl Med. 2012 Aug 22;10:172. doi: 10.1186/1479-5876-10-172 (PMC3479070; doi:10.1186/1479-5876-10-172)

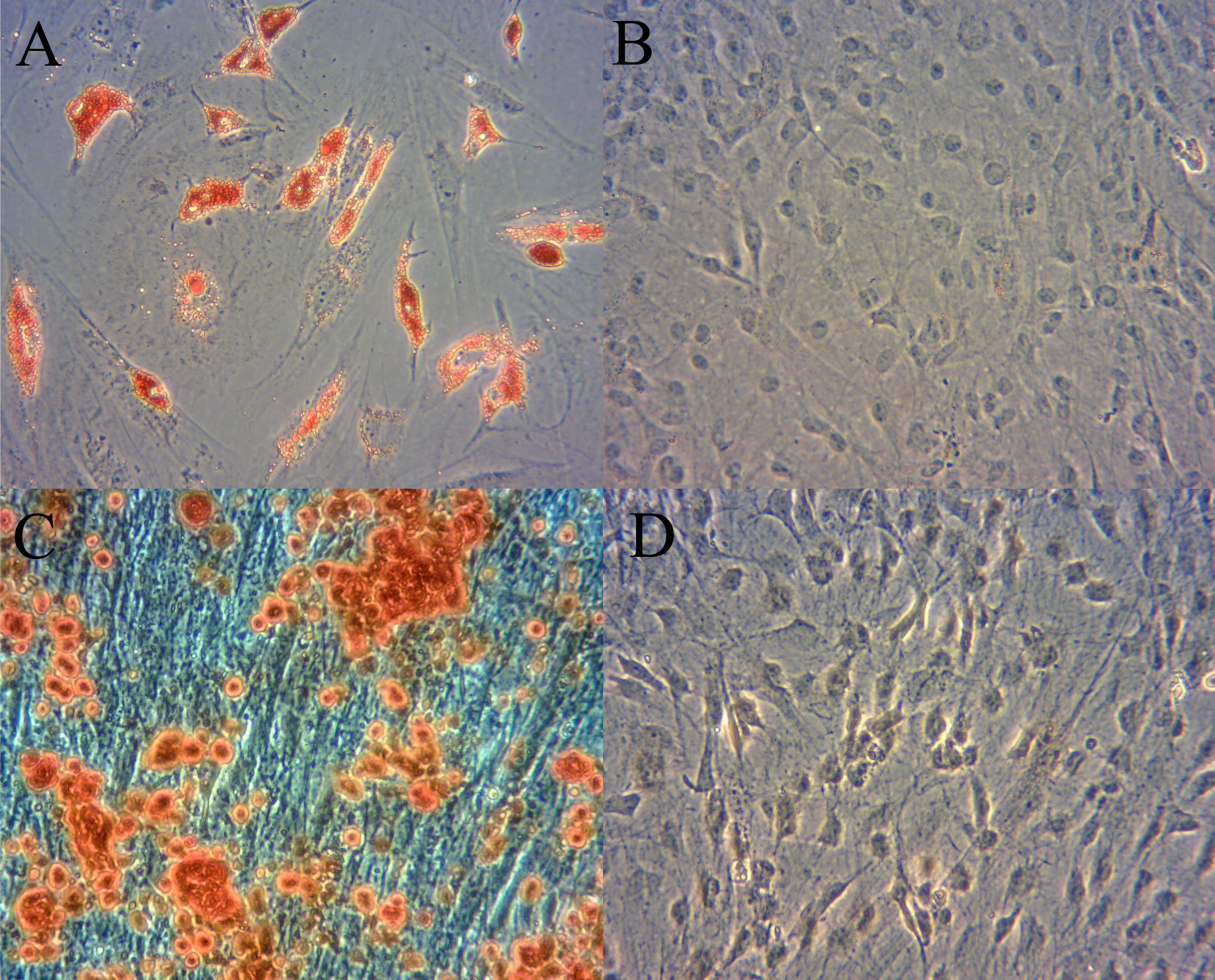

Supplement: Additional file 1 — Figure S1. Differentiation of adherent human adipose-derived cells into adipogenic and osteogenic lineages. Adherent cells were obtained from human lipoaspirate samples and treated with standard differentiation media or control media. Adipogenic differentiated (A) and control (B) cells were stained with Oil Red O to visualize lipid accumulation. Osteogenic differentiated (C) and control (D) cells were stained with Alizarin Red to visualize calcium deposition. [file 1479-5876-10-172-S1.jpeg]
